# Practice of skin protection and skin care among German surgeons and influence on the efficacy of surgical hand disinfection and surgical glove perforation

**DOI:** 10.1186/1471-2334-14-315

**Published:** 2014-06-10

**Authors:** Julian C Harnoss, Laura Brune, Jörg Ansorg, Claus-Dieter Heidecke, Ojan Assadian, Axel Kramer

**Affiliations:** 1 Department of General, Visceral and Transplantation Surgery, University of Heidelberg, Heidelberg 69120, Germany; 2 Institute for Hygiene and Environmental Medicine, Universitätsmedizin Greifswald, Greifswald 17475, Germany; 3 Professional Board of German Surgeons, Berlin 10117, Germany; 4 Department of Surgery, Universitätsmedizin Greifswald, Greifswald 17489, Germany; 5 Clinical Institute for Hospital Hygiene and Infection Control, Medical University of Vienna, Waehringer Guertel 18-20, Vienna 1090, Austria

**Keywords:** Hand disinfection, Surgical hand rub, Skin protection, Skin care, Compliance surgeon, Interaction, Alcohol-based hand rub, Micro-perforation, Surgical glove

## Abstract

**Background:**

Surgical hand rub and healthy skin are basic requirements to prevent surgical site infections. Nevertheless, there is little knowledge about the current practice of skin protection and/or skin care products (SP/SC) using among surgeons as well as a lack of data pertaining to the influence of SP/SC on the antimicrobial efficacy of surgical hand rub.

**Methods:**

A 10 weeks-survey among German surgeons as well as an experimental crossover study involving 26 participants were conducted. The immediate and sustainable efficacy (IE/SE) of surgical hand rub and participants’ hand moisture were measured after an 8-day usage of SP/SC, as well as the influence on micro-perforations on surgical gloves.

**Results:**

The questionnaire was available to 16,000 German surgeons. Thereof, 1,771 surgeons accessed the questionnaire, representing a total participation rate of 11%. As 19% (n = 338) of questionnaires were incomplete, a total of 1,433 completed questionnaires were available for further analysis. More than 75% of the participants stated not to use any SP/SC, yet, almost 50% suffered from skin irritation or discomfort. Only 5% used SP/SC at the beginning of their shift. 10% refused to use SP/SC because of concerns that SP/SC may reduce the antimicrobial efficacy of surgical hand rub.

After usage of SP/SC over 8-days, skin moisture was significantly higher (P < 0.001), whereas no significant influence on the antimicrobial efficacy of surgical hand rub was observed (IE: P = 0.135; SP: P = 0.681). Micro-perforations were detected in 8/52 surgical gloves (15%), with no statistical significant difference between SP/SC users (n = 2/26; 8%) and non-users (n = 6/26; 23%; P = 0.249).

**Conclusions:**

Following the results of this largest questionnaire base survey among German surgeons on skin care, there is a need to educate and inform surgeons on the correct application and the concept of SP/SC strategies. In the present study, the combination of selected SP/SC products and one alcohol-based hand rub formulation did not show a negative interaction with surgical hand rub or surgical glove perforation. However, it is advisable to ascertain the compatibility of SP/SC products with the used hand disinfectant prior to purchase.

## Background

The skin of hands is exposed to a number of physical and chemical substances during hand washing, routine hand disinfection and wearing of gloves, which may reduce the natural protective mechanisms of the skin [1-5]. Healthy skin is a prerequisite for effective hand hygiene, since open wounds and damaged skin may impair disinfection [6]. The efficacy of skin protection and/or skin care products (SP/SC) has been demonstrated in various studies and is therefore recommended for daily use [7-10]. Skin protection (SP) creams shall be used at the beginning of the shift and typically a second time after the lunch break to avoid chemical substances to irritate and penetrate the skin [11]. During the working day and at the end the usage of skin care (SC) products support skin regeneration by replenishing lipids. The consequent application of effective products preserves unimpaired skin, reduces the incidence of skin irritations and dryness and ensures effective hand hygiene [12]. However, there are concerns that SP or SC products may interfere with alcohol-based hand rub formulations, and hence, reduce the efficacy of hand disinfection before surgical procedures [13].

In a previously published prospective questionnaire based survey [14] it was demonstrated that the knowledge on this topic among medical and surgical nurses in a German university medical center was insufficient, leading to wrong behavior at work and inadequate use of SP and SC products. Surprisingly, there is a lack of data pertaining the usage of SP/SC products in the daily clinical routine of practicing surgeons. Little is known if surgeons may exclude the usage of SP/SC products because of concerns of a potential negative influence on the efficacy of hand disinfection prior to surgery. Therefore, the objective of this study was to evaluate the frequency and modality of usage of SP/SC products among surgeons and to investigate the efficacy of hand disinfection under regularly application of SP/SC usage in a longitudinal experimental setting.

## Methods

To evaluate the usage of SP/SC by surgical staff in the daily clinical routine a questionnaire containing nine short questions was designed. Three questions aimed at the person: (1) gender, (2) age, (3) and profession (surgeon/operative technical assistant/nurse). The following 6 questions addressed SP/SC practices and possible skin irritations. Specifically the following was asked:

(4) Is the difference between SP and SC known to you?

(5) Do you start at the beginning of your shift with SP cream, SC cream, or none of both?

(6) How many times daily do you use SP/SC: never, 1-2 times, 3-4 times, >4 times, other (how often?)

(7) Do you use SP/SC (multiple answers are allowed): before every surgical hand disinfection, after hand washing/after operation, 1x in the morning or evening, irregularly after hand washing or surgical hand disinfection, when hands feel rough and dry, only if I have spare time, or not at all?

(8) If you do not use any SP/SC, please indicate why (multiple answers are allowed): time wasting overhead, stress, uncomfortable feeling of the hands after application of SP/SC, unpleasant smell of SP/SC, no SP/SC are available at the workplace, doubt regarding the efficacy of surgical hand disinfection, doubt regarding the efficacy of SP/SC?

(9) Do you suffer from skin irritation (multiple answers are allowed): not any skin irritations, pruritus of the hands, reddening/rubor of the hands, rough and/or dry feeling of the hands, contact dermatitis, finger nail fissures or splittering, other reason.

The Professional Board of German Surgeons (PBGS) sent the digitally created questionnaire via email to all surgical departments in Germany to reach all of the 16,000 registered surgeons. Replying the answers was possible through the website of the PBGS. Only questionnaires returned within 10 weeks after submission were included into the further analysis.

### Experimental study and ethical statement

Additionally to the questionnaire-based survey, a prospective experimental study involving healthy adult participants was conducted. Inclusion and exclusion criteria, randomization, SP/SC, and the parameters to identify benefits or negative influences in the treatment group were defined in a written study protocol approved by the ethics committee of the University of Greifswald according to the Helsinki Declaration (ethic committee votum no. BB18/12). Written informed consent for participation in the study was obtained from all participants.

Twenty-six participants without any visible or diagnosed skin irritations were randomly assigned to one of two study arms, consisting of 13 participants each. Group A started 8 days before the experimental day (ED) 1 and used SP cream (TwinProtect, Precutan®, Evonik Stockhausen GmbH, Krefeld, Germany) and SC products (Cream Sensitive, Precutan®, Evonik Stockhausen GmbH, Krefeld, Germany) three times daily. The participants were instructed to assure the compliance with the recommended order in which the products should be used. The simultaneous usage of other skin products was not allowed. Participants in group A used SP cream one hour before surgical hand rub.

**Figure 1:**
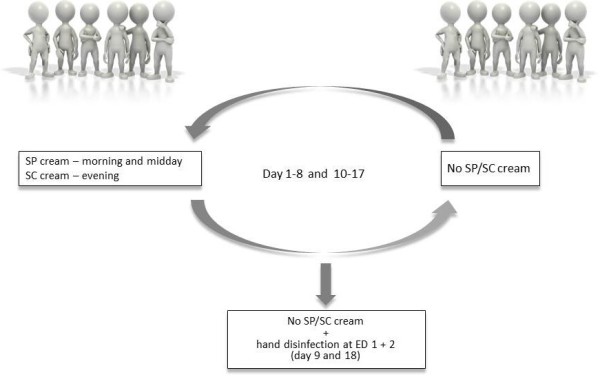
SP/SC: Skin Protection and/or Skin Care products; ED: Experimental Day.

Group B did not use any SP/SC products. At ED 1, the efficacy of surgical hand rub using an alcohol-based hand rub (Sterillium®, Bode Chemie GmbH, Hamburg, Germany) was determined for all participants in both study arms.According to the crossover design, the following day group B started to apply SP/SC three times daily for eight days, while group A had to wait for a minimum of 8 days before the cross-over experiment without SC/SP products was performed. This measure allowed diminishing of the previous SC/SP product application. At ED 2, the efficacy of surgical hand rub again was determined for all participants in both study arms (Figure 1).

At each ED, the skin moisture at three standardized measure points at the back of both hands was measured using a calibrated corneometer (Corneometer®, Courage + Khazaka electronic GmbH, Köln, Germany). These measurements resulted in relative values ranging from 0 to 120. The corneometer values were interpreted as follows: < 30 very dry skin; 30-40 dry skin; and > 40 well moisture skin [15].

In order to standardize surgical hand rub each volunteer received 5 ml of alcohol-based hand rub into both dry palms and rubbed them sufficiently over 90 seconds. First, the forearms were rubbed for 5 seconds. During the next 80 seconds the hands were disinfected following to the European Norm EN 12791 for testing surgical hand disinfection [16]. To measure the efficacy of hand rub the immediate effect (IE) was determined by rubbing the fingertips in a Casein-pepton-Soja-pepton solution (CSL) and transferring a dilution on a Casein-pepton-Soja-pepton agar plate (CSA). A validated neutralizing agent, a mixture of 3% Tween 80 (Merck; Germany), 3% saponin (Riedel-deHaen; Germany), 0.1% histidine hydrochloride (Merck; Germany) and 0.1% cysteine (Merck; Germany) was included into the sampling fluid, but not in the counting plates. During the following three hours sterile surgical gloves were worn (Gammex®, Ansell Healthcare, Melbourne, Australia). Thereafter, the same sampling method was used to determine the 3-hour sustained effect (SE) of hand rub.

Additionally, the frequency of surgical glove micro-perforation was measured following to the European Norm EN 455-1 [17], including assessment of individual glove fingers. One pair of surgical glove was collected from each participant once immediately after wearing gloves during a period of 3-hours in a laboratory. A total of 52 surgical gloves were available to be tested for possible micro-perforations. While the EN 12791 requires wearing sterile surgical gloves under rest conditions over a period of 3-hours without mechanical stress in order to protect participants’ hands form exogenous contamination, we modified the method such that participants were allowed to continue usual office and laboratory work. The reason for this modification was to test possible glove perforation during mechanical stress, which always exists during any surgical procedure. Although the mechanical stress during office and laboratory work does not simulate exactly the forces, which are found during surgical procedures, this measure may yield more realistic results than observing glove perforation after complete rest position.

### Statistical analysis

Statistical analysis and plots were performed using Prism 5 (GraphPad Software, Inc.). Data were presented as median with 1^st^ and 3^rd^ quartile, if not indicated otherwise. Fisher’s exact test was applied for categorical variables. A Wilcoxon rank-sum test or Kruskal-Wallis test, as indicated, was used for quantitative data. A statistically significant difference was assumed when *P* was less than or equal to 5% (*P* ≤ 0.05).

## Results

The questionnaire was available to all 16,000 surgeons currently listed with the PBGS in Germany. Thereof, 1,771 surgeons accessed the questionnaire and submitted their replies. Hence, the total participation rate was 11%, which represents a large proportion of German surgeons. As 19% (n = 338) of questionnaires were incomplete, a total of 1,433 completed questionnaires were available for further analysis.

The majority of respondents were practicing surgeons (98%, n = 1,405). More than three quarters of participants were male (77%, n = 1,108), and most of them were 41-50 years old (41%, n = 588). 38% (n = 537) of respondents stated having regularly dry and rough feeling of the hands, 14% (n = 196) stated having broken finger nails or fissures, 12% (n = 178) pruritus, and 10% (n = 145) rubor of the hands. Half of the respondents (49%, n = 702) stated not to suffer from any skin irritation.

More than half of the respondents (57%, n = 817) stated to know the differences between SP and SC products. However, at the beginning of the day 78% (n = 1,115) do not apply SP/CP at all, 14% (n = 197) used SC products, and 5% (n = 75) SP cream. During the complete working shift, 47% (n = 681) never use SP/SC routinely, 40% (n = 571) once to twice daily, and 9% (n = 132) three or more times. Mostly, SP/SC are only used when the hands feel rough and dry (36%, n = 515), at the beginning or at the end of the shift (22%, n = 312), and randomly during the day (21%, n = 296), respectively.

SP/CP is not used for several reasons: 27.6% (n = 396) disliked the hand feeling or smell, 23.9% (n = 343) indicated stress at their working places as the main reason and even 10.2% (n = 146) had concerns that SP/SC may impair the efficacy of surgical hand rub.

### Experimental study

In both study arms, the measured skin moisture was significantly higher after SP/SC (group A: 43.2 ± 11.83 vs. group B: 34.5 ± 11.83; P = 0.0006), Table 1. Moist condition of hands improved from "very dry" to "dry", however, in total not reaching a "well moisture" condition.

**Table 1:** Relative hand moisture with/ without SP/SC usage

**Skin moisture**	**Min.**	**Max.**	**Volunteers (n)**	**1**^**st **^**Quartile**	**Median**	**3**^**rd **^**Quartile**
Without SP/SC	21.67	51.00	26	29.17	34.50	41.00
With SP/SC	29.17	106.50	26	35.84	43.17	47.67

Corneometer® relative values: < 35 very dry, 35-50 dry and > 50 sufficiently wet; SP/SC: Skin Protection and/ or Skin Care products; P=0.0006 (Fisher’s exact test, Wilcoxson rank-sum test or Kruskal-Wallis test, as appropriated).

Without using any SP/SC products, the immediate (IE) and sustained (SE) bacterial reduction factors (log_10_) were 2.8 ± 1.49 (IE) and 1.57 ± 2.4 (SE), respectively. After application of SP/SC during 8 consecutive days, the bacterial reduction factors (log_10_) were 1.98 ± 1.83 (IE) and 1.84 ± 1.41 (SE), respectively. The application of SP/SC product had no significant influence on the efficacy of hand disinfection (IE: P = 0.135; SE: P = 0.681). Detailed results are summarized in Table 2.

**Table 2:** **IE/ SE log**_**10 **_**cfu count after hand rub with/ without SP/SC usage**

	**Min.**	**Max.**	**Volunteers (n)**	**1**^**st **^**Quartile**	**Median**	**3**^**rd **^**Quartile**
IE without SP/SC	0.52	5.25	26	1.74	2.80	3.23
IE with SP/SC	-0.54	4.75	26	1.41	1.98	3.24
SE without SP/SC	-0.06	4.69	26	0.73	1.57	3.13
SE with SP/SC	-0.04	5.28	26	1.26	1.84	2.67

IE: Immediate Effect; SE: Sustained Effect; SP/SC: Skin Protection and/ or Skin Care products; IE: P=0.137; SE: P=0.681 (Fisher’s exact test, Wilcoxson rank-sum test or Kruskal-Wallis test, as appropriated).

In total, micro-perforation was detected in 8/52 surgical gloves (15%) of which 6 occurred in participants not using SP/SC products during 8 consecutive days before surgical hand rub, and 2 in participants using SP/SC products. Although trend-wise the frequency of micro-glove perforation was higher in participants without usage of SP/SC products, the difference in micro-perforation within the SP/SC group (2/26, 7.7%) and non-SP/SC group (6/26, 23.1%) was statistically not significant (P = 0.249, two-tailed Fisher’s exact test).

## Discussion

To our knowledge, this survey is the largest ever performed on practices of skin care or skin protection practices among surgeons. However, one of the limitations of the results may be that particularly surgeons with personal interest in hand hygiene or those with chronic or intermitted skin disorders may have responded. This may have introduced a participation bias, distorting results towards higher knowledge on SP/SC application or higher proportions in reported skin affections. Indeed, about half of the respondent stated not having any form of skin irritation, and more than half stated to know the difference between SP and SC products. Even if a participation bias may be present in the collected data, the fact that chief criteria such as self-reported knowledge and skin condition are distributed evenly among a large proportion of German surgeons allows drawing meaningful conclusions.

While it is difficult to state that surgeons reporting having knowledge on SP and SC, the opposite may certainly be assumed. In fact, some 40% of the responding surgical staff did not know the difference between SP and SC products. This may result in incorrect usage. Furthermore, among the 57% of the respondents stating to be familiar with SP/SC products, only 5% used SP cream in the morning, as recommended by the manufacturers. Consequently, training and educational programs still seem to be necessary to explain the importance of SP products among surgical professionals [8,10]. This is underlined by the response of 36% of the respondents stating to use SP/SC when the hands already feel rough and dry. On one side, this may indicate that existing skin irritations markedly increase the willingness to use SP/SC products; on the other side, the strength of SP products is to prevent skin affections in the first place, and hence, their usage after manifested skin conditions is only the second best strategy.

One factor, which may inhibit the primary usage of SP products, is their smell, as 35% of respondents disliked the smell and the feeling on hands after SP product usage. Improved SP formulations may increase the primary usage of SP products before skin irritations may develop. Most likely, easily absorbing, odorless, and perfume-free products should be preferred.

Some 10% of respondents stated that they have concerns that previous use of SP/SC products may negatively affect the efficacy of surgical hand rub. Indeed, this view sometimes is also shared in hand hygiene or surgical site infection prevention sessions during surgical conferences. While infrequently discussed, there is little data to support or reject this view. Therefore, we conducted an experimental crossover study among practicing surgeons. For the intervention arm, we chose highly absorbing, odorless, and perfume-free SP/SC products to exclude possible influences of added ingredients. Furthermore, a SC cream with a low urea concentration was selected in order to increase skin permeability [18] and to avoid increased absorption of the alcohol during surgical hand disinfection [19,20]. The alcohol-based hand rub Sterillium® was selected as it is the product with the highest market share in Germany and therefore may be representative best for many surgical departments. Following the European Norm EN 12791 and in order to avoid contaminating of hands during a 3-hour period, all participants wore identical sterile surgical gloves of a premium brand with an AQL of 0.065. Finally, to control for a number of influencing factors, all experiments were conducted during the same season and daytime, excluding participants with visible skin damage to avoid insufficient hand disinfection [21].

The results of this study did not show any significant differences for the efficacy of surgical hand disinfection, regardless of usage of SP/SC products. These results are in line with a previous study investigating the possible influence of SP cream on hand disinfection among nurses in medical and surgical intensive care [14]. However, some older in-vitro studies showed decreased antimicrobial efficacy of hand disinfection when SP/SC products were used [22-24]. Based on these studies the Association of Professionals in Infection Control and Epidemiology (APIC) recommended ascertaining the compatibility of SP/SC products with the used hand disinfectant [25] prior to purchase. While reasonable, it has to be noted that these almost 20-30 year old studies had investigated the influence on liquid antimicrobial hand wash soaps containing chlorhexidine gluconate. In large parts of Europe, however, traditionally alcohol-based hand rubs are used, with or without chlorhexidine gluconate content. Therefore, it remains difficult to draw general conclusions from one study or series of studies exploring different products and formulations, and the APIC Guideline Committee’s recommendations to ascertain the products’ compatibility still shall be followed. Regretfully, for the majoritiy of SP, SC, and hand rub products such information is not available, as the possible effects were not tested or not published.

Our study clearly demonstrated that after regular use of SP/SC products over a period of one week, skin moisture significantly increased. However, based on the corneometry measurements the skin of hands still was "dry" and did not reach a well moisture condition, which is indicated by corneometry measurements above 40 points. Furthermore, this relative improvement was reached only without regular surgical hand preparation, including hand washing with liquid soap, which may further dehydrate the skin of [26,27]. In this context, it is important to note that the skin of participants’ hands were "dry" (corneometry measures [15] ranging between 30 - 40 points) with a median of 34.5 points. Hence, if hands are dry and not cared for, it may not be expected that a well moisture state will be achieved within one week, but it requires longer usage of SP/SC products to improve the condition of the affected skin.

An interesting side finding of our experimental study was that a higher proportion of micro-perforations was detected in participants that did not use SP/SC products prior to surgical hand disinfection. Although the difference in micro-perforation between those using SP/SC products (7.7%) 8 days prior to glove wear and those, which did not use SP/SC products (23.1%) was statistically not different – which may be the result of a possible beta error due to small observation numbers – an overall glove micro-perforation rate of 15% may seem to be surprising. The observed grove perforation rate in our present study was almost 15x times higher than what may have been expected at rest conditions [28]. As our experimental study was designed as a crossover study with 26 participants included to test both conditions, with and without prior use of SP/SC products under identical conditions, neither the selected SP/SC products, the alcohol-based hand rub or the type of surgical gloves, which were of premium quality with a lower than average AQL of 0.065 as compared to some other surgical gloves with lower quality, may serve as sole and conclusive explanation for the observed difference.

One factor remaining as possible explanation for the difference in glove perforation is the improved skin condition of participants during wearing of gloves. Indeed, although not statistically different, the glove perforation rate in participants which had used SP/SC products one week prior to the surgical hand disinfection and wear of gloves over a 3-hour period at rest was 7.7%, while in participants without use of SP/SC products, the perforation rate was 23.1%. Since concurrently with the usage of SP/SC products the moisture condition of the skin of hands significantly improved, it may be speculated that dry hands and cracks in skin may lead to increased micro-perforation of surgical gloves, even at rest. Although our study yields data that may support this view, this study was not designed to study in detail the correlation of dry skin and glove perforation rates. However, this aspect may warrant further studies in the operation theatre.

## Conclusions

Following the results of the largest questionnaire base survey among German surgeons on skin care, there is a need to educate and inform surgeons on the correct application and the concept of SP/SC strategies. Any doubts raised by surgeons regarding the possibility of interaction of SP/SC products with the efficacy of surgical hand disinfection should be taken seriously. In the present study, the combination of selected SP/SC products and one alcohol-based hand rub formulation did not show a negative interaction. However, it is advisable to ascertain the compatibility of SP/SC products with the used hand disinfectant prior to purchase.

## Abbreviations

APIC: Association of Professionals in Infection Control and Epidemiology; AQL: Acceptable quality level for surgical gloves (GAMMEX AQL: 0.065); cfu: Colony-forming unit; ED: Experimental day; IE: Immediate efficacy of surgical hand rub; PBGS: Professional Board of German Surgeons; SC: Skin care; SE: Sustainable efficacy surgical hand rub; SP: Skin protection.

## Competing interests

This study was financed through the routine research grant of the Institute for Hygiene and Environmental Medicine, University Medicine Greifswald, Germany. The authors have no competing interests to report. A Kramer and O Assadian have partly received travel compensation and speakers honoraria from Bode Chemie GmbH in the past. O Assadian is member of the Medical Scientific Advisory Board of Hutchinson santé, France.

## Author’s contributions

JC Harnoss, A Kramer, and CD Heidecke planned and designed the experimental study. JC Harnoss, A Kramer, CD Heidecke, and J Ansorg planned and designed the questionnaire. JC Harnoss, A Kramer, and L Brune supervised and coordinated data collection. JC Harnoss, A Kramer, and O Assadian performed the statistical analysis. All authors have participated in analysis and interpretation of data, drafting and revising the manuscript, and have read and approved to the final version of the manuscript.

## Pre-publication history

The pre-publication history for this paper can be accessed here:

http://www.biomedcentral.com/1471-2334/14/315/prepub

## References

[B1] di Nardo A Sugino K Wertz P Ademola J Maibach HI Sodium lauryl sulfate (SLS) induced irritant contact dermatitis: a correlation study between ceramides and in vivo parameters of irritation Contact Derm 1996 35 86 91 10.1111/j.1600-0536.1996.tb02296.x 8917825

[B2] Gfatter R Hackl P Braun F Effects of soap and detergents on skin surface pH, stratum corneum hydration and fat content in infants Dermatology 1997 195 258 262 10.1159/000245955 9407174

[B3] Kirk JE Hand washing. Quantitative studies on skin lipid removal by soaps and detergents based on 1500 experiments Acta Derm Venereol 1966 46 Suppl 57 1 183 5282609

[B4] Kramer A Mersch-Sundermann V Gerdes H Pitten FA Tronnier H Kampf G Toxikologische Bewertung für die Händedesinfektion relevanter antimikrobieller Wirkstoffe Hände-Hygiene im Gesundheitswesen 2003 Berlin: Springer 105 160

[B5] Warner RR Stone KJ Boissy YL Hydration disrupts human stratum corneum ultrastructure J Invest Dermatol 2003 120 275 284 10.1046/j.1523-1747.2003.12046.x 12542533

[B6] Mäkelä P Kramer A, Weuffen W, Gröschel D, Heeg P, Hingst V, Lippert H, Rotter M Gesunde Haut als Vorraussetzung für eine effektive Händedesinfektion Klinische Antiseptik 1993 New York: Springer 97 103

[B7] Szepietowski JC Salomon J Hand dermatitis among nurses: the reasons and consequencies Contact Derm 2006 54 129 130 10.1111/j.0105-1873.2006.00568.x 16487294

[B8] Fluhr JW Gloor M Lehmann L Lazzerini S Distante F Berardesca E Glycerol accelerates recovery of barrier function in vivo Acta Derm Venereol 1999 79 418 421 10.1080/000155599750009825 10598752

[B9] Kutting B Baumeister T Weistenhofer W Pfahlberg A Uter W Drexler H Effectiveness of skin protection measures in prevention of occupational hand eczema: results of a prospective randomized controlled trial over a follow-up period of 1 year Brit J Derm 2010 162 362 370 10.1111/j.1365-2133.2009.09485.x 19804591

[B10] Löffler H Bruckner T Diepgen T Effendy I Primary prevention in health care employees: a prospective intervention study with a 3-year training period Contact Derm 2006 54 202 209 10.1111/j.0105-1873.2006.00825.x 16650095

[B11] Mahler V Skin protection in the healthcare setting Curr Prob Dermatol 2007 34 120 132 10.1159/000099993 17312362

[B12] Smith DR Leggat PA Hand dermatitis among female nursing students in tropical Australia Nurs Health Sci 2004 6 109 113 10.1111/j.1442-2018.2004.00181.x 15130096

[B13] Grinbaum RS deMendonca JS Cado DM An outbreak of handscrubbing- related surgical site infections in vascular surgery procedures Infect Control Hosp Epidemiol 1995 16 198 202 10.2307/30140978 7636166

[B14] Große-Schütte K Assadian O Hübner NO Löffler H Kramer A Practices of skin care among nurses in medical and surgical intensive care units: results of a self-administered questionnaire GMS Krankenhaushyg Interdiszip 2011 6 20111215 Doc08 10.3205/dgkh000165 PMC3252660 22242089

[B15] Heinrich U Koop U Leneveu-Duchemin MC Osterrieder K Bielfeldt S Chkarnat C Degwert J Häntschel D Jaspers S Nissen HP Rohr M Schneider G Tronnier H Multicentre comparison of skin hydration in terms of physical-, physiological- and product-dependent parameters by the capacitive method (Corneometer CM 825) Int J Cosmet Sci 2003 25 45 53 10.1046/j.1467-2494.2003.00172.x 18494882

[B16] EN 12791 Chemical Disinfectants and Antiseptics. Surgical Hand Disinfection. Test Method and Requirement (Phase 2, Step 2) 2005 Brussels: European Committee for Standardization

[B17] EN 455-1 Medical Gloves for Single Use 2000 Brussels: European Committee for Standardization

[B18] Cahill JL Nixon RL Allergic contact dermatitis in health care workers to diazolidinyl urea present in antimicrobial hand gel Med J Aust 2011 194 664 665 21692731 10.5694/j.1326-5377.2011.tb03161.x

[B19] Kramer A Below H Bieber N Kampf G Toma CD Hübner NO Assadian O Quantity of ethanol absorption after excessive hand disinfection using three commercially available hand rubs is minimal and below toxic levels for humans BMC Infect Dis 2007 7 117 10.1186/1471-2334-7-117 17927841 PMC2089071

[B20] Below H Partecke I Hübner NO Bieber N Nicolai T Usche A Assadian O Below E Kampf G Parzefall W Heidecke CD Zuba D Bessonneau V Kohlmann T Kramer A Dermal and pulmonary absorption of propan-1-ol and propan-2-ol from hand rubs Am J Infect Control 2012 40 250 257 10.1016/j.ajic.2011.03.009 21741120

[B21] Forrester BG Roth VS Hand dermatitis in intensive care units J Occup Environ Med 1998 40 881 885 10.1097/00043764-199810000-00008 9800173

[B22] Benson L LeBlanc D Bush L White J The effects of surfactant systems and moisturizing products on the residual activity of a chlorhexidine gluconate handwash using a pigskin substrate Infect Control Hosp Epidemiol 1990 11 67 70 10.2307/30144264 2179400

[B23] Larson E Anderson JK Baxendale L Bobo L Effects of a protective foam on scrubbing and gloving Am J Infect Control 1993 21 297 301 10.1016/0196-6553(93)90386-I 8122801

[B24] Walsh B Blakemore PH Drabu YJ The effect of handcream on the antibacterial activity of chlorhexidine gluconate J Hosp Infect 1987 9 30 33 10.1016/0195-6701(87)90091-0 2880895

[B25] APIC Guideline Committee APIC guideline for handwashing and hand antisepsis in healthcare setting Am J Infect Control 1995 23 251 269 10.1016/0196-6553(95)90070-5 7503437

[B26] Hübner NO Kampf G Kamp P Kohlmann T Kramer A Does a preceding hand wash and drying time after surgical hand disinfection influence the efficacy of a propanol-based hand rub BMC Microbiol 2006 6 57 10.1186/1471-2180-6-57 16790073 PMC1513577

[B27] Hübner NO Kampf G Löffler H Kramer A Effect of a 1 minute hand wash on the bactericidal efficacy of consecutive surgical hand disinfection with standard alcohols and on skin hydration Int J Hyg Environm Health 2006 209 285 291 10.1016/j.ijheh.2006.01.002 16488665

[B28] Partecke LI Goerdt AM Langner I Jaeger B Assadian O Heidecke CD Kramer A Huebner NO Incidence of microperforation for surgical gloves depends on duration of wear Infect Control Hosp Epidemiol 2009 30 409 414 10.1086/597062 19335225

